# Metabolomic Changes Associated with AGXT2 Genotype Variants and Stone Formation in a Colony of Cats

**DOI:** 10.3390/genes15101264

**Published:** 2024-09-27

**Authors:** Jean A. Hall, Jeffrey A. Brockman, John J. Brejda, Dennis E. Jewell

**Affiliations:** 1 Department of Biomedical Sciences, Carlson College of Veterinary Medicine, Oregon State University, Corvallis, OR 97331, USA; 2 Science & Technology Center, Hill′s Pet Nutrition, Inc., Topeka, KS 66617, USA; jeff_brockman@hillspet.com; 3 Alpha Statistical Consulting, Inc., Lincoln, NE 68502, USA; alphastatistical01@gmail.com; 4 Department of Grain Science and Industry, Kansas State University, Manhattan, KS 66506, USA; djewell@ksu.edu

**Keywords:** calcium oxalate stones, cats, genomics, metabolomics, serum

## Abstract

Objective: The objective of this study was to assess serum chemistries and metabolomic parameters in cats with genetic variants of the alanine-glyoxylate aminotransferase 2 (AGXT2) gene to determine abnormalities associated with urolith formation and better understand effective approaches for the treatment of cats with uroliths. Methods: AGXT2 genotypes of 445 cats in the colony at Hill’s Pet Nutrition, Inc. (Topeka, KS, USA) were assessed in a genome-wide association study. Additionally, the serum chemistries and metabolic profiles of each cat were determined, along with their lifetime history of stone incidence. Factor analysis was used as a data-reduction method for metabolites in order to perform statistical hypothesis testing and to select significant metabolites from the more than 600 serum metabolites identified. Results: Of the 82 cats forming stones in the colony (18.4%), the majority were calcium oxalate. Results showed that approximately one third of the cats with the AA variant of the AGXT2 gene have stones, that chronic kidney disease (CKD) is more common in cats with stones, and that having stones results in a shorter lifespan. A discriminant variable selection process was performed to determine the complete blood count, serum biochemistries, and serum metabolomic factors that best discriminated among the three genotypes (AA, AG, GG) and between cats forming stones and non-stone formers. Several of the highly ranked discriminating factors included metabolites related to decreased aminotransferase activity in cats with the AA variant of the AGXT2 gene. Another factor that ranked highly for discriminating between stone formers and non-stone formers contained lipid metabolites, consisting of multiple sphingomyelin species and cholesterol. Conclusions: These findings support the results of feeding studies in cats, whereby CKD cats fed food supplemented with betaine and prebiotics have experienced an increase in total body mass, reduced uremic toxins, and altered sphingomyelin concentrations.

## 1. Introduction

We previously reported that the lifespan of cats with non-obstructive kidney stones is shorter compared with stone-free geriatric cats, suggesting that kidney stones have an effect on the mortality rate and/or rate of chronic kidney disease (CKD) progression [1]. However, the true incidence of urolithiasis in cats is unknown as they may go undiagnosed unless there are clinical signs of lower urinary tract disease, including urethral obstruction [2]. For example, urolithiasis accompanies 15 to 20% of cats with clinical signs of lower urinary tract disease [3,4].

The predominant composition of uroliths varies depending on the study, from predominantly calcium oxalate (CaOx; 68.8%) followed by struvite (24.2%) in the Netherlands [5] to predominantly struvite (54.4%) followed by CaOx (37.7%) in California, USA [6]. Although guessing the composition of uroliths is unreliable [2], with nutritional management, certain stones can be dissolved and the risk of recurrence reduced or prevented [7]. For example, a struvite dissolution diet in cats with struvite urolithiasis is highly effective in dissolving struvite uroliths within 30 to 70 days and is suitable as a maintenance food for the long-term prevention of feline struvite urolithiasis [8]. Because cats in the colony at Hill’s Pet Nutrition, Inc. (Topeka, KS, USA) are fed struvite calculolytic and preventative commercial foods, the majority of uroliths observed in the colony cats are CaOx.

Nutritional management to dissolve CaOx uroliths is generally not successful [2,7]. In addition, dietary acidification does not change the risk of CaOx crystallization [9]. In most cats with CaOx uroliths, the underlying etiopathogenesis is unknown [2]. Some breeds of cats are predisposed to CaOx uroliths, including Burmese and Persian in one study [6], British shorthair, Burmese, Persian, Radoll, or Tonkinese in another study [10], and all cat breeds except Domestic Shorthair in a third study [5], suggesting a genetic component. Hypercalciuria appears to be more important than the urine oxalate concentration in determining crystal formation [2,11,12]. 

In humans, calcium-based calculi account for approximately 75% of kidney stone disease, and the incidence is increasing, suggesting environmental (dehydration) and dietary factors (high dietary animal protein and sodium content) are acting upon the genetic predisposition [13]. Heritability is estimated at 46 to 56%, with males > females [14]. Gene-wide association studies (GWAS) in different populations have identified multiple associated single nucleotide polymorphisms, or SNPs, of a moderate-to-little effect size (OR 1.2 to 2.1), including solute transporters, chloride channels, tight junction proteins, and metabolizing enzymes [14]. These GWASs suggest a series of genes involved in the renal tubular handling of calcium, oxalate, and phosphate and inhibitors of crystallization, such as citrate and magnesium [13]. Hypercalciuria is the prevailing metabolic trait [14]. 

In order to develop effective approaches for the treatment of CaOx uroliths in cats, genetic and metabolic abnormalities that promote urolith formation need to be identified [2]. For example, we recently showed that cats in the colony at Hill’s Pet Nutrition having the most common variant (GG) of alanine-glyoxylate aminotransferase 2 (AGXT2) respond positively to a dietary intervention known to reduce the risk of CaOx stone formation compared with cats having the AA or AG variants [12]. The purpose of the current study was to evaluate all 445 cats in the Hill’s Pet Nutrition colony to identify the complete blood count (CBC), serum chemistry, and serum metabolite parameters associated with the AA, AG, and GG genotypic variants of the AGXT2 gene in cats. In addition, CBC, serum chemistry, and serum metabolite findings associated with CaOx uroliths diagnosed either ante- or postmortem were identified. 

## 2. Materials and Methods

All study protocols were reviewed and approved by the Institutional Animal Care and Use Committee, Hill’s Pet Nutrition, Inc., Topeka, KS, USA (Permit Number: CP815.1.2.0-A-F-D-ADH-MULTI-84-KID), and complied with the National Institutes of Health Guide for the Care and Use of Laboratory Animals [15]. A colony of random bred domestic-short-hair cats (*n* = 445) was housed in groups and allowed access to indoor runs. Cats also had exposure to natural light that varied with seasonal changes. All cats were provided with regular opportunities to exercise, with access to toys. Cats were owned by the commercial funders of this research or their affiliates, who gave permission for them to be included in this study. At the conclusion of the study, all cats were returned to the Hill’s Pet Nutrition, Inc. colony.

### 2.1. Serum Metabolomics 

Blood was collected from each cat (after withholding food for 17 h; food was withheld at the end of the day, and blood was drawn the next morning) over a 6-week period in April and May of 2016. The analysis of serum metabolomic profiles was performed by a commercial laboratory (Metabolon, Morrisville, NC, USA) as previously described [16]. Briefly, the extracted supernatant was split and run on gas chromatography and liquid chromatography mass spectrometer platforms in randomized order. Gas chromatography (for hydrophobic molecules) and liquid chromatography (for hydrophilic molecules) were used to identify and provide the relative quantification of small metabolites present in serum samples. Endogenous biochemicals included amino acids, peptides, carbohydrates, lipids, nucleotides, cofactors, and vitamins.

### 2.2. Gene-Wide Association Study 

The cats in the colony at Hill’s Pet Nutrition, Inc. were genotyped on a custom cat iSelect Illumina high-density genotyping array with 340,000 attempted bead types, as previously reported [12]. Approximately 272,000 SNPs gave reliable genotype calls. Genotypes were converted to population-based linkage analysis (PLINK) format, and quality control was performed using PLINK v1.9 (https://www.cog-genomics.org/plink2, accessed 25 September 2024) [17,18]. After filtering (genotype calls > 0.90, maf > 0.05), 437 individuals and 194,151 SNPs remained in the dataset.

### 2.3. Complete Blood Count and Serum Biochemistries

Blood collected from each cat was also used to assess CBC and serum biochemistries. Urine was not collected at the same time as blood was collected for metabolomics, GWAS, CBC, and serum biochemistries, but rather was collected once a year at the time of the performance of annual examinations. Therefore, urine parameters are not included in this report.

### 2.4. Stone Formation and CKD Diagnosis

Stone formation was documented ante mortem after finding stones on radiographs or post mortem at necropsy up until March 2024. Stones were located in the kidneys or urinary bladder. Not all stones diagnosed ante mortem were found at necropsy. A diagnosis of CKD was made using International Renal Interest Society criteria (IRIS Stages 1 to 4) [19]. A diagnosis of CKD was also documented up until March 2024.

### 2.5. Statistical Methods

Statistical analyses were performed using SAS version 9.4 (SAS Institute, Cary, NC, USA). Forty-three CBC and serum biochemistry analytes were included in the analysis. Each of these analytes was subjected to an analysis of variance (ANOVA) with genotype (AA, AG, GG), stones (present or absent), and the interaction as fixed-effects in the model. Because this was an exploratory study, no control for inflation of the type I error rate was used. 

More than 600 serum metabolites were included in the analysis (**Supplemental Table S1**). However, many of these metabolites are correlated because they serve as precursors, intermediate products, or end products for other metabolites. To take advantage of this correlation, factor analysis was used as a data-reduction method for the metabolites [20,21]. For this study, principal component analysis was used as the method of factor extraction. The resulting patterns were then rotated using a varimax rotation to aid interpretation. The advantage of a varimax rotation is that all of the factors remain orthogonal (independent) and each metabolite tends to load highly on only one factor, making it easier to interpret the factor patterns. Factor analysis was performed on the correlation matrix so that unequal variances among the metabolites did not unduly influence the resulting factor patterns. Using the correlation matrix, each metabolite has a variance of 1. To reduce the number of factors that needed to be interpreted, only factors with eigenvalues greater than 6 were retained for rotation. An eigenvalue of 6 indicates that a factor accounts for at least 1% of the total variation in the data. Using this approach, the 600 serum metabolites were reduced to 20 factors (Supplemental Table S2). The resulting factors were then analyzed using ANOVA as described above with genotype, stones, and the interaction as fixed-effects in the model. Factor means are linear combinations of all metabolites, with higher coefficients or weights for metabolites that load strongly on that factor and small coefficients or weights for metabolites that are only weakly or not associated with that factor. Because the serum metabolic data are median-normalized, a negative mean indicates that the mean level of the metabolic factor is below the median, and a positive mean indicates that the mean level of that factor is above the median. 

PROC STEPDISC was used to select the CBC, serum biochemistry, and serum metabolomic factors that discriminated among the three genotypes (AA, AG, GG). The STEPDISC procedure performs a stepwise discriminant analysis to select a subset of the CBC, serum biochemistry, and serum metabolite factors for use in discriminating among the genotypes [20,21]. With the STEPDISC procedure, the METHOD = STEPWISE, PR2ENTRY = 0.01, and PR2STAY = 0.01 options were used. The STEPWISE method performs stepwise variable selection such that the variable that contributes the most to the discriminatory power of the model is entered. This is continued sequentially until all variables that meet the entry and stay requirements are met. The PR2ENTRY option specifies the partial R^2^ for adding variables in the model. The PR2STAY option specifies the partial R^2^ for retaining variables in the model. The criterion of 0.01 was selected because the chosen and retained variables must account for at least 1% of the variation in the data. The discriminant analysis described above was repeated to select the CBC, serum biochemistry, and serum metabolomic factors that discriminated between cats that formed stones and those that did not. 

## 3. Results

### 3.1. Genome-Wide Association Study of Colony Cats

As previously reported [12], the GWAS of 445 cats in the colony at Hill’s Pet Nutrition, Inc. revealed a 1.6 megabase region on cat chromosome A1 (chrA1) with a strong association with serum concentrations of the metabolite 2-oxoarginine. The coding sequence for the AGXT2 gene is centered in this region (located between base pair positions 210,631,461 and 212,243,690 [NCBI Felis Catus genome assembly 6.2/felcat5]). The most significant SNP associated with 2-oxoarginine in this region was at position chrA1:212069607, [G/A] (*p* < 3.687 × 10^−17^). This SNP explained approximately 15% of the variance in 2-oxoarginine serum concentrations in this cohort of cats. The distribution of genotype frequencies across the cohort of cats was 0.07 AA, 0.39 AG, and 0.54 GG, with a mean relative serum 2-oxoarginine concentration for each genotype of 0.45 AA, 0.92 AG, and 1.27 GG, indicating an additive effect of the minor allele (A).

There was no difference in genotypes based on gender (*p* = 0.37) and no difference in cats with stones based on gender (*p* = 0.97) (**Table 1**; **Supplemental Table S3A,B**). There was a statistically significant difference in genotypes based on age if all cats in the study were included (*p* = 0.038), with cats having the AA genotype being on average the youngest and cats with the GG genotype the oldest. This difference in genotypes based on age was only a trend if only cats that had died were included (*p* = 0.09). There was no difference in genotypes based on the CKD diagnosis (*p* = 0.56). There was no difference in the presence/absence of stones based on age if all cats in the study were included (*p* = 0.46). If only cats that had died were included in the analysis, then there was a difference between stone-formers and non-stone-formers in that stone-formers died 1 year younger (12.03 vs. 13.08 years) than non-stone-formers (*p* = 0.02). Of the cats with the AA genotype, 33% had stones vs. 17% of cats with the AG, and 18% of cats with the GG genotypes had stones (*p* = 0.09 Pearson Chi-Square). Cats that formed stones had a higher incidence of CKD. (Of the stone-forming cats in the database, 33/82 had CKD (40.2%) vs. cats that did not form stones, where only 66/363 (18.2%) had CKD (*p* < 0.0001 Pearson Chi-Square). In the 82 cats forming stones (18.4% of cats in the colony), 58 stones were available for analysis (16 cats are still alive with stone analysis pending and 8 deceased cats had no stone analysis performed): 50 were calcium-containing stones (44 were CaOx), 5 were struvite, and 3 were 100% miscellaneous.

### 3.2. CBC and Serum Biochemistries

The CBC and serum biochemistry analytes that varied significantly among the three genotypes were the mean corpuscular volume (MCV), absolute reticulocyte count, neutrophil %, blood urea nitrogen (BUN), and symmetric dimethylarginine (SDMA). The CBC and serum biochemistry analytes that varied significantly between cats that did or did not have stones were the mean corpuscular hemoglobin (MCH), MCV, hematocrit (HCT), hemoglobin, total protein, albumin:globulin ratio, alkaline phosphatase (ALP), BUN, cholesterol, globulin, inorganic phosphorus, creatinine, glucose, and SDMA. The CBC and serum biochemistry analytes for which there was a significant interaction among genotype and the presence or absence of stones were BUN and creatinine. Significant results are summarized in Table 2. All results are included in **Supplemental Table S4**.

Concentrations of SDMA were higher in cats with the AA genotype and lower in cats with the GG genotype with either the presence or absence of stones. Cats with the AA genotype and stones had the highest SDMA concentrations, and cats with the GG genotype and no stones had the lowest SDMA concentrations. Creatinine concentrations were higher in cats of all genotypes with stones compared with cats without stones. There was an interaction for genotype*stone such that cats with AA or AG genotypes with stones had a higher creatinine increase compared with cats having the GG genotype and stones. The BUN concentration was similarly higher in cats of all genotypes with stones, and there was an interaction for genotype*stone such that the presence of stones caused a higher BUN in cats with the AA or AG genotypes. Globulin concentrations were higher in cats with stones compared with cats without stones, and the corresponding albumin:globulin ratio was thereby lower in cats with stones. Alkaline phosphatase and inorganic phosphorus concentrations were lower in cats of all genotypes if stones were present. Cholesterol, glucose, and total protein concentrations were higher in cats with stones from all genotypes. For CBC analytes, the hemoglobin concentration, hematocrit percentage, and MCH were lower in cats of all genotypes if stones were present. The MCV was lower in cats of the AA genotype compared with cats of the GG genotype, whereas the absolute reticulocyte count was higher in cats having the AA genotype. The % neutrophils was lower in cats with the AA genotype compared with cats having the GG genotype, although absolute neutrophil numbers were not significantly different based on the genotype.

### 3.3. Serum Metabolite Factors

Serum metabolite factors that varied among the three genotypes were Factor 2, Factor 3, Factor 12, Factor 18, and Factor 20. Serum metabolite factors that varied significantly between cats that did or did not have stones were Factor 1, Factor 3, Factor 5, Factor 9, and Factor 12. There was a significant interaction between the genotype and presence or absence of stones for Factor 3. Significant factors are summarized in Table 3. All serum metabolites were reduced to 20 factors and are included in **Supplemental Table S2**. All statistical results for serum metabolite factors are included in **Supplemental Table S5**.

Metabolites in the Factor 3 group were significant for genotype and for the presence of stones in that values were higher for cats having the AA genotype and higher in the presence of stones. There was an interaction for genotype*stone such that cats with AA or AG genotypes with stones had a greater Factor 3 influence compared with cats having the GG genotype. Metabolites in the Factor 12 group were also significant for genotype and for the presence of stones in that values were higher for cats having the AA genotype and higher in the presence of stones. Metabolites for Factor 2, Factor 18, and Factor 20 were all significant for genotype in that values were highest for cats having the GG genotype and lowest for cats having the AA genotype, regardless of the presence/absence of stones. Metabolites for Factor 1 were significant for the presence of stones in that values were higher for cats having stones regardless of genotype. Metabolites for Factor 5 and Factor 9 were also significant for the presence of stones, but these values were lower for cats having stones regardless of the genotype.

### 3.4. Discriminant Variable Selection among Genotypes

The STEPDISC procedure selected SDMA as the single analyte with the greatest discriminatory power among genotypes, with a partial R^2^ of 0.0726. SDMA was significantly higher with genotype AA (14.5 ± 0.6 μg/dL) compared with the genotype AG (12.7 ± 0.2 μg/dL) or GG (12.1 ± 0.2 μg/dL). SDMA means for AG and GG were similar. 

After SDMA, the STEPDISC procedure selected cholesterol as the second analyte that best discriminated among the three genotypes, with a partial R^2^ of 0.0234. The ANOVA indicated there was no significant difference (*p* = 0.09) among the three genotypes for cholesterol. Cholesterol, however, was numerically lower with genotype AA (145 ± 11 mg/dL) compared with either genotype AG (149 ± 4 mg/dL) or GG (162 ± 3 mg/dL). 

After SDMA and cholesterol, the STEPDISC procedure selected Factor 3 as the third variable for discriminating among the three genotypes, with a partial R^2^ of 0.0188. Factor 3 had large positive loadings for N6-carbamoylthreonyladenosine, pseudouridine, allantoin, N1-methylinosine, C-glycosyltryptophan, N-acetyltaurine, allantoic acid, erythronate, 7-methylguanine, erythritol, N-acetylthreonine, O-sulfo-L-tyrosine, dimethylarginine (ADMA + SDMA), 1-methylhistidine, N-acetylserine, and kynurenate and moderate negative loadings for docosadioate and threonine. The ANOVA indicated a statistically significant difference among the three genotypes for Factor 3. Factor 3 means were positive or above the median values for genotype AA (0.14 ± 0.22) but negative or below the median values for genotypes AG (−0.10 ± 0.08) and GG (−0.08 ± 0.07). 

After Factor 3, the STEPDISC procedure selected Factor 12 as the fourth variable for discriminating among the three genotypes, with a partial R^2^ of 0.0204. Factor 12 had large positive loadings for pyruvate, α-ketoglutarate, lactate, fumarate, malate, 3-methyl-2-oxobutyrate, alanine, 2-oxoadipate, 4-methyl-2-oxopentanoate, 3-methyl-2-oxovalerate, N-acetylglutamine, and 2-hydroxy-3-methylvalerate and a moderate negative loading for 4-guanidinobutanoate. The ANOVA indicated that Factor 12 means were significantly different among the three genotypes. The mean for Factor 12 was higher for genotype AA (0.09 ± 0.22) compared with genotypes AG (0.04 ± 0.08) and GG (−0.12 ± 0.07).

After Factor 12, the STEPDISC procedure selected Factor 18 as the fifth variable for discriminating among the three genotypes, with a partial R^2^ of 0.0177. Factor 18 had large positive loadings for cysteine sulfinic acid, benzoate, and heme; moderate positive loadings for biliverdin, cortisone, pyridoxal, 2’-deoxyuridine, and glycerol; a large negative loading for mannitol/sorbitol; and moderate negative loadings for maltose and thioproline. The ANOVA indicated that Factor 18 was significantly lower or below the median for genotype AA (−0.42 ± 0.22) compared with genotypes AG (0.06 ± 0.08) and GG (0.03 ± 0.07). 

After Factor 18, the STEPDISC procedure selected total bilirubin as the sixth variable for discriminating among the genotypes, with a partial R^2^ of 0.0146. The ANOVA indicated there was no significant difference (*p* = 0.34) among the three genotypes for total bilirubin. Total bilirubin, however, was numerically highest with genotype AA (0.047 ± 0.021 mg/dL) compared with genotypes AG (0.031 ± 0.008 mg/dL) and GG (0.044 ± 0.007 mg/dL).

After total bilirubin, the STEPDISC procedure selected Factor 1 as the seventh variable for discriminating among the genotypes, with a partial R^2^ of 0.0158. Factor 1 was primarily composed of sphingomyelins. Rather than list all of them here, the reader is referred to Table 3A. The ANOVA indicated that Factor 1 means did not vary significantly among the three genotypes (*p* = 0.07). However, the Factor 1 mean for genotype AA was numerically much more negative or below the median values (−0.43 ± 0.22) than the means for AG (−0.08 ± 0.08) and GG (−0.02 ± 0.07). 

After Factor 1, the STEPDISC procedure selected Factor 20 as the eighth variable for discriminating among the genotypes, with a partial R^2^ of 0.0137. Factor 20 had moderate positive loadings for γ-glutamylfelinylglycine, sulfate, 7-hydroxyindole sulfate, cysteine s-sulfate, homocitrulline, isovalerylglycine, 2-oxoarginine, 4-guanidinobutanoate, fumarate, dimethylmalonic acid, and malate and a moderate negative loading for N-linolenoyltaurine. Factor 20 varied significantly among the three genotypes (*p* = 0.0006). Factor 20 mean was numerically negative or below median values for genotypes AA (−0.28 ± 0.22) and AG (−0.09 ± 0.08) but positive for GG (0.05 ± 0.07). 

After Factor 20, the STEPDISC procedure selected Factor 2 as the ninth variable for discriminating among the genotypes, with a partial R^2^ of 0.0118. Factor 2 was composed of several carnitine metabolites that all had large positive loadings on this factor. Rather than list all of them here, the reader is referred to Table 3A. Factor 2 varied significantly among the three genotypes (*p* = 0.04). Factor 2 means were negative (below the median value) for genotypes AA (−0.12 ± 0.22) and AG (−0.18 ± 0.08) and positive (above the median value; 0.15 ± 0.07) for GG. 

After Factor 2, the STEPDISC procedure selected Factor 5 as the tenth variable for discriminating among the three genotypes, with a partial R^2^ of 0.0107. Factor 5 had large positive loadings for linoleate (18:2n6), oleate/vaccenate (18:1), palmitate (16:0), stearate (18:0), 10-nonadecenoate (19:1n9), eicosenoate (20:1n9 or 20:1n11), margarate (17:0), palmitoleate (16:1n7), 10 heptadecenoate (17:1n7), dihomolinolenate (20:3n3 or 20:3n6), dihomolinoleate (20:2n6), and nonadecanoate (19:0) and moderate negative loading for p-cresol sulfate, tryptophan, and hypotaurine. The ANOVA indicated that Factor 5 did not vary significantly among the three genotypes (*p* = 0.43). Numerically, the Factor 5 mean was higher for genotype AA (0.39 ± 0.22) compared with AG (0.16 ± 0.08) and GG (−0.07 ± 0.07). 

After Factor 5, the STEPDISC procedure selected glucose as the eleventh variable for discriminating among the three genotypes, with a partial R^2^ of 0.0104. The ANOVA indicated there was no significant difference (*p* = 0.48) among the three genotypes for glucose. 

No other CBC, serum biochemistry, or serum metabolite factors had partial R^2^ values that met the criteria for inclusion in the model for discriminating among genotypes. All of the 11 variables listed above were retained in the final model. The results from the discriminant analysis are summarized in Table 4, including the order that they were selected, their partial R^2^ values, and statistical significance. Not all variables are statistically significant (i.e., cholesterol, total bilirubin, Factor 1, Factor 5, and glucose) because the partial R^2^ option was used to select variables, not p-values. 

### 3.5. Discriminant Variable Selection for Stone Formation

The STEPDISC procedure selected Factor 3 as the variable that had the greatest discriminatory power between stone forming and non-stone-forming cats, with a partial R^2^ of 0.0260. The metabolites with high loadings on Factor 3 were discussed previously for discrimination among genotypes. Factor 3 was significantly higher for stone-formers compared with non-stone-formers with the AA (0.66 ± 0.31 vs. 0.14 ± 0.22) and AG (0.70 ± 0.19 vs. −0.10 ± 0.08) genotypes. For the GG genotype, there was very little difference among stone-formers (0.02 ± 0.15) and non-stone-formers (−0.08 ± 0.07). 

After Factor 3, the STEPDISC procedure selected alkaline phosphatase as the second variable that best discriminated between stone-formers and non-stone-formers, with a partial R^2^ of 0.0205. Alkaline phosphatase was significantly (*p* = 0.04) lower for stone-forming cats vs. non-stone-formers regardless of the genotype: AA (27.9 ± 7.4 vs. 36.5 ± 5.4 U/L), AG (24.8 ± 4.4 vs. 32.5 ± 2.0 U/L), and GG (23.5 ± 3.5 vs. 29.6 ± 1.7 U/L).

After alkaline phosphatase, the STEPDISC procedure selected MCHC as the third variable that best discriminated between stone-formers and non-stone-formers, with a partial R^2^ of 0.0233. The ANOVA indicated there was no significant difference (*p* = 0.06) between stone-formers and non-stone-formers. MCHC, however, was numerically higher regardless of the genotype for stone-formers vs. non-stone-formers: AA (33.7 ± 0.5 vs. 33.1 ± 0.4 g/dL), AG (33.4 ± 0.3 vs. 33.0 ± 0.1 g/dL), and GG (33.2 ± 0.2 vs. 32.9 ± 0.1 g/dL).

After MCHC, the STEPDISC procedure selected Factor 1 as the fourth variable that best discriminated between stone-formers and non-stone-formers, with a partial R^2^ of 0.0160. However, based on the partial R^2^, it was not as important as Factor 3. The ANOVA indicated that Factor 1 varied significantly between stone-forming cats and non-stone-formers. With non-stone-formers, Factor 1 had large negative mean scores with the AA genotype (−0.43 ± 0.22), indicating below median concentrations, and mean scores very close to 0 with the AG (−0.08 ± 0.08) and GG (−0.02 ± 0.07) genotypes. With stone-formers, Factor 1 had a negative mean score for the AA genotype (−0.14 ± 0.31) but positive means scores for the AG (0.28 ± 0.19) and GG (0.41 ± 0.15) genotypes.

After Factor 1, the STEPDISC procedure selected total protein as the fifth variable that best discriminated between stone-formers and non-stone-formers, with a partial R^2^ of 0.0120. The ANOVA indicated that total protein was significantly (*p* = 0.02) higher for stone-forming cats vs. non-stone-formers regardless of the genotype: AA (7.0 ± 0.2 vs. 6.8 ± 0.1 g/dL), AG (7.2 ± 0.1 vs. 6.9 ± 0.05 g/dL), and GG (7.0 ± 0.1 vs. 6.9 ± 0.04 g/dL).

After total protein, the STEPDISC procedure selected magnesium as the sixth variable that best discriminated between stone-formers and non-stone-formers with a partial R^2^ of 0.0116. The ANOVA indicated that there was no significant difference (*p* = 0.46) for magnesium between stone-formers and non-stone-formers.

After magnesium, the STEPDISC procedure selected sodium as the seventh variable that best discriminated between stone-formers and non-stone-formers, with a partial R^2^ of 0.0103. The ANOVA indicated that there was no significant difference (*p* = 0.25) for sodium between stone-formers and non-stone-formers. 

No other CBC, serum biochemistry, or serum metabolite factors had partial R^2^ values that met the criteria for inclusion in the model for discriminating between stone-formers and non-stone-formers. All of the seven variables listed above were retained in the final model. The results from the discriminant analysis are summarized in Table 5, including the order that they were selected, their partial R^2^ values, and statistical significance. Not all variables were significant (MCHC, magnesium, sodium) because the partial R^2^ option was used to select variables, not *p*-values. The reason that ANOVA and discriminant function results may differ is that the ANOVA looks at each variable independently or one at a time. It does not account for or adjust for information about other variables in the dataset. In contrast, the discriminant analysis looks at partial correlations between the class variable (stone formation) and the predictor variables after adjusting for all other variables in the dataset. Thus, it looks at variables as a group, not independently, and adjusts for other variables. 

## 4. Discussion

### 4.1. Genotype and Stone Formation Demographics

The purpose of this study was to identify CBC, serum biochemistry, and metabolomic factors associated with the AA, AG, and GG genotype variants of the AGXT2 gene, along with factors associated with stone formation in cats. All 445 cats in the Hill’s Pet Nutrition colony were studied. Based on gender, we found no difference in genotype or stone formation. Based on the age at death (lifespan) of cats in the study, those with stones died one year younger on average, 12 vs. 13 years of age, compared with cats without stones. There was a trend for cats with the AA genotype to die at an earlier age (*p* = 0.09). We previously reported that the lifespan of cats with non-obstructive kidney stones is shortened compared with healthy cats indicating a need to prevent stone formation and minimize CKD [1]. In the former study, cats with kidney stones were diagnosed from August 2010 to December 2015 (n = 43). The cats in the current study represent a subsequent population of cats with kidney stones (n = 82). 

There was no difference in CKD based on the genotype, but cats that formed stones (18% of cats in the colony) had a significantly higher incidence of CKD (40.2% of stone formers had CKD vs. 18.2% of non-stone-formers). The majority of stones were calcium-containing (44 were CaOx, 5 were struvite, and 3 were miscellaneous) in the 58 cats that had stones available for analysis. We were unable to show a significant relationship between stone formation and genotype (*p* = 0.09) likely because of the small number of AA cats having stones. Cats with the least common genetic variant of the AGXT2 gene (AA; 6.7% of cats in colony) had a numerically greater incidence of stones (33.3% had stones), compared with cats having the AG (37.5% of cats in colony; 16.8% had stones) or GG variant (55.7% of cats in colony; 17.7% had stones). Previously, we have shown that cats having the GG variant respond positively to a dietary intervention that reduces the risk of CaOx stone formation compared with cats having the AA or AG variants [12]. We conclude that the majority of stones in the cat colony are CaOx, that approximately one third of the cats with the AA variant of the AGT2 gene have stones, that CKD is more common in cats with stones, and that having stones results in a shorter lifespan. 

### 4.2. Complete Blood Count and Serum Biochemistry Analytes

The CBC and serum biochemistry analytes that were found to discriminate among the three genotypes were the MCV, absolute reticulocyte count, neutrophil %, BUN, and SDMA. The MCV was lower in cats with the AA genotype but especially lower in cats with stones and AA genotype. A low mean corpuscular volume is characteristic of iron-deficiency anemia, although mean values for HCT (%) and RBC counts were within normal reference intervals for cats. It is likely that individual cats with stones suffered iron depletion from chronic blood loss in the urine, leading to microcytic, hypochromic anemia. The absolute reticulocyte count was significantly higher in cats with the AA genotype and numerically highest in cats with the AA genotype and stones, indicating somewhat regenerative anemia. The SDMA was higher in cats with the AA genotype in both stone-formers (highest) and non-stone-formers. Cats with the GG genotype had lower SDMA concentrations in both stone-formers and non-stone-formers (lowest). The renal biomarker SDMA is increased in cats with CKD, and CKD was more common in cats with stones. We have shown previously that using serum SDMA as a biomarker for CKD allows for the earlier detection of CKD compared with serum creatinine, which may be desirable for initiating renoprotective interventions that slow the progress of CKD [22]. Conversely, BUN was highest in cats with GG and AG genotypes compared with the AA genotype and lowest in cats forming stones with the GG genotype. Cats with AG or AA genotypes and stones had similar BUN. The percent neutrophils was higher in cats with AG and GG genotypes compared with the AA genotype, whereas the absolute neutrophil count was highest in cats with the AG genotype and stones, with an interaction between the genotype and stone formation.

In addition to MCV, SDMA, and BUN, the serum biochemistry analytes that were found to discriminate between stone-formers and non-stone-formers were hematocrit (%), hemoglobin, MCH, total protein, globulin, albumin:globulin ratio, cholesterol, inorganic phosphorus, ALP, creatinine, and glucose. The mean corpuscular hemoglobin, hemoglobin concentration, and HCT were lower in cats with stones compared with cats with no stones, again likely as a result of microscopic hematuria associated with stones and urinary tract infections. The BUN was higher in all cats with stones. This was likely because CKD was more common in cats with stones compared with non-stone formers and BUN is a biomarker for CKD. However, there was a significant interaction for stones and genotype such that cats with the AA genotype had higher BUN concentrations compared with cats having the GG genotype, a finding similar to SDMA concentrations in cats with stones and different genotypes. Similar to BUN, the serum creatinine concentration was also higher in cats with stones and highest with cats of the AA genotype. Serum total protein and globulin concentrations were higher in cats with stones and consequently resulted in a lower albumin:globulin ratio. Increased globulin suggests chronic inflammation consistent with chronic urolithiasis, and in humans, a decreased albumin:globulin ratio serves as a prognostic factor for predicting postoperative febrile urinary tract infection after lithotripsy [23]. Glucose values were higher in cats with stones and cats with the AA genotype, and stones had the highest glucose values. It is difficult to determine whether hyperglycemia preceded urolithiasis or urolithiasis resulted in hyperglycemia. In humans, there is epidemiological evidence that supports diabetes mellitus as a risk factor for the development of kidney stone disease [24].

Cholesterol was higher in all cats with stones. In a longitudinal study involving humans that was designed to investigate the risk of dyslipidemia and kidney stone disease, after adjusting for age, sex, smoking and alcohol history, diabetes, hypertension, fasting glucose, albumin, and eGFR, researchers demonstrated that hypertriglyceridemia, low high-density lipoprotein cholesterol (HDL-C), and a high Chol/HDL-C ratio were associated with a higher risk of developing kidney stones [25]. In another study, although higher triglyceride and HDL-C concentrations were found in stone-formers, lower LDL-C and total cholesterol concentrations were also reported in patients with stones compared to those without stones [26]. Nonetheless, it is clear for humans that dyslipidemias may play a role in urinary stone risk. Hypercalciuria and hyperoxaluria occur in rat animal models with hypertriglyceridemia and hypercholesterolemia [27]. In dogs, although hypertriglyceridemia and hyperglycemia were associated with uroliths, cholesterol concentrations were not different between dogs with uroliths and urolith-free dogs [28]. 

Serum ALP and inorganic phosphorus were both lower in cats with stones. Altered phosphorus homeostasis may contribute to urolithiasis [29]. Several studies have shown lower values of serum phosphorus and a higher fractional excretion of phosphate in stone-forming human patients [29]. It is possible that higher plasma fibroblast growth factor 23 (FGF23), an osteocyte-derived phosphaturic hormone, may contribute to CaOx stone formation [29,30]. In the kidney, FGF-23 acts on sodium-phosphorus type II co-transporters to reduce proximal tubular capacity to reabsorb phosphate ions, thereby increasing phosphate ion loss in the urine [31]. We recommend future studies to assess serum FGF-23 concentrations and the fractional excretion of phosphorus in cats with stones.

### 4.3. Serum Metabolomic Factors 

Biological inferences from >600 metabolites are difficult without applying complex data analysis methods. Principal component analysis (PCA) is commonly used to visualize metabolomic data in a two-or three-dimensional space. The eigenvectors for autoscaled data in PCA [32] are proportional to the correlation coefficients between the PC scores and the variables [20]. The eigenvector is associated with a set of linear equations and identifies the directions of maximum variation in the data. The eigenvalue is the amount of variation in each direction. Factor loading is defined as the correlation coefficients between the PC scores and the variables [33]. Factor loading is used to perform statistical hypothesis testing and to select significant metabolites objectively using statistical criteria. By choosing only factors with eigenvalues > 6, such that the factors account for at least 1% of total variation in the data, the 600 serum metabolites in this study were reduced to 20 factors. These 20 factors were then analyzed using ANOVA with genotype, stones, and the interaction as fixed-effects in the model. Individual serum metabolite factors that that were found to discriminate among the three genotypes were Factor 2, Factor 3, Factor 12, Factor 18, and Factor 20. Individual serum metabolite factors that were found to discriminate between stone-formers and non-stone-formers were Factor 1, Factor 3, Factor 5, Factor 9, and Factor 12. Because Factor 3 and Factor 12 appeared in both analyses, we decided to focus on metabolites clustered within these two factors.

The goal of the discriminant variable selection process was to determine the CBC, serum biochemistries, and serum metabolomic factors that best discriminated among the three genotypes (AA, AG, GG) and between cats forming stones and non-stone-formers. The stepwise method chooses the variable that contributes the most to the discriminatory power of the model and continues sequentially until all variables that meet the entry and stay requirements are met. Options for both were set at 0.01 such that chosen and retained variables had to account for at least 1% of the variation in the data. This resulted in ten discriminant variables selected among genotypes and six discriminant variables selected between stone- and non-stone-formers. Among the variables selected for discriminating among the AA, AG, and GG genotypes and the variables selected for discriminating between stone-formers and non-stone-formers, two were common to both analyses—Factor 3 and Factor 1. Factor 12 ranked highly (right after Factor 3) in variables selected for discriminating among the AA, AG, and GG genotypes. None of the CBC or serum biochemistry analytes were present in both analyses. Thus, for the purposes of this discussion, we chose to focus on Factor 3 and Factor 12, as mentioned above, as well as Factor 1.

A number of Factor 3 metabolites are found in nucleotide pathways (N6-carbamoylthreonyladenosine, pseudouridine, allantoin, N1-methylinosine, allantoic acid, 7-methylguanine), and thus, they are involved in purine and pyrimidine metabolism. The means were highest in stone-forming cats with the AA genotype. A number of Factor 3 metabolites were also amino acids involved in tryptophan metabolism (C-glycosyltryptophan, kynurenate), threonine or serine metabolism (N-acetylthreonine, N-acetylserine), taurine metabolism (N-acetyltaurine), arginine metabolism (ADMA + SDMA), and histidine metabolism (1-methylhistidine). The means were highest in stone-forming cats with the AA genotype. One metabolite in Factor 3 was a carbohydrate involved in aminosugar metabolism (erythronate). Because AGXT2 is a promiscuous, multifunctional, mitochondrial aminotransferase involved in the metabolism of amino acids, as well as in nucleotide catabolism [34], we hypothesized that these elevated metabolite concentrations in cats forming stones were related to decreased aminotransferase activity in cats with the AA variant of the AGXT2 gene. We have previously reported that serum concentrations of two AGXT2 substrates, SDMA/ADMA dimethylarginines and β-aminoisobutyrate (BAIB), the latter being an end product of pyrimidine metabolism, were increased in cats with the AA variant of the AGXT2 gene and that concentrations of 2-oxoarginine were reduced, the latter being a metabolite produced via the deamination of arginine [12]. We also have seen several of the metabolites listed above increased in cats with renal disease or calcium oxalate stone formation in other studies [12,35,36,37,38,39]. 

Factor 12 metabolites included α-keto acids and Kreb cycle intermediates (pyruvate, α-ketoglutarate, lactate, fumarate, malate), the principle amino acid substrate for hepatic gluconeogenesis (alanine), metabolites derived from the incomplete breakdown of branched chain amino acids (3-methyl-2-oxobutyrate, 4-methyl-2-oxopentanoate, 3-methyl-2-oxovalerate), intermediates in the metabolism of lysine and tryptophan (2-oxoadipate), and an essential cofactor for the enzyme that catalyzes the first step of the urea cycle (NAG or N-acetylglutamine). We hypothesized that these elevated metabolite concentrations were also related to decreased aminotransferase activity as AGXT2 is a promiscuous, multifunctional, mitochondrial aminotransferase involved in the metabolism of multiple amino acids [34]. As supporting evidence, Factor 12 means were higher in cats with the AA variant of the AGXT2 gene and higher in cats forming stones (highest in cats with the AA variant and stones). Overall, metabolites for Factor 3 and Factor 12 sorted similarly based on genotype and presence of stones.

Factor 1 metabolites were lipids, primarily sphingomyelins and cholesterol. The Factor 1 mean for genotype AA was numerically much more negative and below the median values (−0.43 ± 0.22) than means for AG (−0.08 ± 0.08) and GG (−0.02 ± 0.07). This was the same for stone-forming cats with the AA genotype, i.e., Factor 1 was negative or below the median values (−0.14 ± 0.31) for cats of the AA genotype but positive and much higher mean scores for AG (0.28 ± 0.19) and GG (0.41 ± 0.15) genotypes (*p* = 0.02). Thus, stones were associated with higher levels of sphingomyelins and cholesterol lipids, although overall concentrations of Factor 1 metabolites were lowest for the variant AA genotype. 

Sphingomyelins and cholesterol are found in animal cell membranes, and in addition to providing structure to the membrane, they also participate in signaling functions. Sphingolipids are a diverse class of lipids with over 4000 distinct species, all characterized by an amino alcohol backbone [40]. As reviewed by Slott [41], the long-chain base can be modified by functional groups to yield, e.g., sphingosine-1-phosphate and ceramide. Ceramide can be further modified to yield sphingomyelin and simple or complex glycosphingolipids. Additional structural variations (hydroxylation, methyl-branching, acyl chain length) allow for even more molecular species within the sphingolipids [41]. 

Sphingolipid metabolites have complex roles in inflammatory signaling pathways, and in turn, their regulation is influenced by inflammatory pathways [40]. Classically, sphingolipids are thought to be second messengers that propagate the inflammatory response. However, dietary sphingolipids may have anti-inflammatory properties. Thus, the regulation of inflammatory processes may be impacted by the balance of sphingolipids, in addition to their abundance [40]. Sphingolipids have a rapid turnover and their concentrations are controlled by the activities of the enzymes involved in their synthesis in the endoplasmic reticulum and Golgi and by degradation in lysosomes (reviewed by Maceyka and Spiegel [42]). 

In this study with cats, we showed that stone formation was associated with higher concentrations of 14 sphingomyelins and cholesterol, although cats with the AA variant of the AGXT2 gene had the lowest concentrations. We have previously shown that feeding CKD cats betaine- and soluble-fiber-supplemented foods resulted in improvements in the body composition and changes in the plasma metabolome that corresponded to better kidney health [38,43]. In both studies, a number of sphingomyelin metabolite concentrations were higher in cats consuming the test food, suggesting that increases in these sphingomyelins may be more reflective of less severe CKD and are perhaps beneficial in delaying CKD progression [38,43].

It is reported that elevated concentrations of oxalate and calcium oxalate crystals provoke renal cells to synthesize inflammatory mediators (reviewed in [44]), likely the result of oxidative stress and the production of free radicals. For example, the exposure of kidney cells in vitro to oxalate increased the production of ceramide, likely through the activation of sphingomyelinase, as many signaling molecules are activated by ROS [44]. At this time, it is unclear how inflammation regulates bioactive sphingolipid concentrations, the activities of the enzymes that control their concentrations, the proteins through which they signal, and how this shapes the immune response [42]. 

In people, hyperlipidemia—either hypertriglyceridemia, hypercholesterolemia, or both—is related to an increased risk of nephrolithiasis. Hypercalciuria and hyperoxaluria occur in rat models with hypertriglyceridemia and hypercholesterolemia. In dogs, serum triglycerides and glucose are associated with uroliths, but cholesterol concentrations are not different between urolith-harboring dogs and urolith-free dogs [28]. In the current study with cats, cholesterol and glucose were significantly increased in cats forming stones, but neither was selected in the stepwise discriminant analysis among CBC, serum biochemistry, and serum metabolomic factors that discriminated between cats that formed stones and those that did not. The exact pathogenesis underlying the association between dyslipidemia and nephrolithiasis remains unclear.

### 4.4. Therapeutic Strategies Based on Genotype and Metabolomic Factors 

We have previously shown that urine from cats with the GG variant of the AGXT2 gene requires more added oxalate to initiate crystal formation after consuming a test food enriched with betaine (0.500%) and the botanicals green tea, fenugreek, and tulsi (0.25, 0.025, and 0.0015%, respectively) [12]. This also corresponded to favorable decreases in the concentrations of some metabolites in Factor 3 and Factor 12 that were associated with stone formation (e.g., N-acetylthreonine, C-glycosyltryptophan, malate) [12]. Thus, dietary management based on the genotype and metabolomic factors for cats with the GG-specific variant of AGXT2 may reduce the risk of calcium oxalate stone formation.

In general, dietary management of cats with CKD results in improved survival (reviewed in [45]). Cats with IRIS stage 1 CKD have lower indoxyl sulfate and p-cresol sulfate concentrations when fed a lower protein diet (6.95 g/100 cal ME or 5.65 g/100 kcal ME) compared with cats fed a higher protein diet (8.01 g/100 kcal ME) [46]. Feeding a test food with potential anti-aging benefits was also shown to result in favorable changes in plasma and fecal metabolites in senior cats [47]. Feeding cats with CKD food that is supplemented with betaine and prebiotics has been showed to increase the total body mass, reduce uremic toxins, and alter sphingomyelin concentrations [38,43]. Dietary betaine also interacts with dietary polyunsaturated fatty acids (PUFAs) to increase concentrations of specific PUFAs, including linoleic acid, arachidonic acid, and the n-3 fatty acids α-linolenic acid and docosahexaenoic acid [48]. The combination of dietary betaine and fish oil reduced circulating 3-indoxyl sulfate concentrations in the cat suggesting a renal benefit from changes in the plasma metabolome [38].

## 5. Conclusions

We found that the majority of stones in this cat colony are CaOx and that approximately one third of the cats with the AA variant of the AGXT2 gene have stones. Thus, decreased aminotransferase activity associated with the AA variant of the AGXT2 gene suggests a genotype predilection and a mechanism, at least in some cats with CaOx stones. CKD was more common in cats with stones, and having stones resulted in at least a one-year shorter lifespan. The discriminant variable selection process was used to determine the CBC, serum biochemistries, and serum metabolomic factors that best discriminated among the three genotypes (AA, AG, GG) and between cats forming stones and non-stone-formers. Factor loading was used to select significant metabolites and revealed that many of the metabolites with increased concentrations in cats forming stones were related to decreased aminotransferase activity in cats with the AA variant of the AGXT2 gene. The presence of stones was also associated with higher levels of sphingomyelins and cholesterol lipids, although overall concentrations of these metabolites were lowest for cats having the AA genotype. The potential for personalized nutrition strategies based on genotype and metabolomic factors is evidenced by studies showing the successful dietary management of cats with CKD. For example [12], we have previously shown that a betaine and botanical dietary enhancement lowers urine oxalate concentrations independent of the genotype, and cats having the GG variant of the AGXT2 gene have a decreased risk of oxalate crystal formation with betaine and botanical dietary enrichment.

## 6. Patents

United States Patent No. US 11,155,856 B2; Date of Patent 26 October 2021. Methods for identifying a companion animal susceptible to treatment that reduces the risk of stone formation and compositions for reducing such risk. Applicant: Hill’s Pet Nutrition, Inc. Inventors: Dennis Jewell, Jeffrey Brockman, Kiran Panickar, and Laura Morgan.

## Figures and Tables

**Table 1:** Chronic kidney disease (CKD) and stone formation in cats with different AGXT2 SNPs from a GWAS of 445 cats in the colony at Hill’s Pet Nutrition, Inc. Also shown are gender and age comparisons.

Genotype	Normal	CKD	Total	Prob > Χ^2^		Genotype	Neutered Male	Spayed Female	Total	Prob > Χ^2^
**AA, n**	25	5	30	0.5592		**AA, n**	15	15	30	0.3706
%	83.3	16.7				%	50.0	50.0		
**AG, n**	126	41	167			**AG, n**	84	83	167	
%	75.5	24.6				%	50.3	49.7		
**GG, n**	195	53	248			**GG, n**	108	140	248	
%	78.6	21.4				%	43.6	56.5		
**Total**	346	99	445			**Total**	207	238	445	
										
**Genotype**	**Normal**	**Stones**	**Total**	**Prob > Χ^2^**		**StoneFormer**	**Neutered Male**	**Spayed Female**	**Total**	**Prob > Χ^2^**
**AA, n**	20	10	30	0.0899		**No, n**	169	194	363	0.9719
%	66.7	33.3				%	46.6	53.4		
**AG, n**	139	28	167			**Yes, n**	38	44	82	
%	83.2	16.8				%	46.3	53.7		
**GG, n**	204	44	248			**Total**	207	238	445	
%	82.3	17.7								
**Total**	363	82	445							
		
**Age of All Cats in the Study (Alive and Dead)**										
**Genotype**	**n**	**Mean Age (y)**	**SE**	**Prob > F**		**StoneFormer**	**n**	**Mean** **Age (y)**	**SE**	**Prob > F**
AA	30	11.26	0.50	0.0378		No	363	12.43	0.14	0.4601
AG	167	12.29	0.21			Yes	82	12.18	0.30	
GG	248	12.58	0.17							
										
**Age of Cats that had Died**										
**Genotype**	**n**	**Mean Age (y)**	**SE**	**Prob > F**		**StoneFormer**	**n**	**Mean** **Age (y)**	**SE**	**Prob > F**
AA	19	11.38	0.68	0.0899		No	181	13.08	0.22	0.0196
AG	87	12.91	0.32			Yes	59	12.03	0.39	
GG	134	12.97	0.26							

**Table 2:** CBC and serum biochemistry analytes that varied significantly among the three genotypes in cats with different AGXT2 SNPs from a GWAS of 445 cats in the colony at Hill’s Pet Nutrition, Inc. Although the mean corpuscular hemoglobin concentration (MCHC), magnesium, and sodium did not vary significantly, their results are included because MCHC was selected as the third variable for discriminating between stone-formers and non-stone-formers with a partial R^2^ of 0.0233, magnesium was selected as the sixth variable that best discriminated between stone-formers and non-stone-formers with a partial R^2^ of 0.0116, and sodium was selected as the seventh variable that best discriminated between stone-formers and non-stone-formers with a partial R^2^ of 0.0103.

		Stones	Genotype = AA				Genotype = AG				Genotype = GG				*p*-Values	
Analyte			n	Mean	SE		n	Mean	SE		n	Mean	SE		Source	Prob > F
Globulin		No	19	3.453	0.149		137	3.464	0.055		201	3.473	0.046		Genotype	0.5912
(g/dL)		Yes	10	3.780	0.205		28	3.811	0.123		44	3.630	0.098		Stones	0.0070
															Gen*Stones	0.5109
																
Album:Globulin		No	19	1.021	0.054		137	1.020	0.020		201	1.018	0.017		Genotype	0.544
Ratio		Yes	10	0.880	0.074		28	0.911	0.044		44	0.968	0.035		Stones	0.0073
															Gen*Stones	0.4988
																
Alkaline		No	19	36.47	5.39		137	32.46	2.01		201	29.64	1.66		Genotype	0.4918
Phosphatase		Yes	10	27.90	7.43		28	24.79	4.44		44	23.50	3.54		Stones	0.0444
(U/L)															Gen*Stones	0.9531
																
Blood Urea		No	19	20.02	0.99		137	20.76	0.37		201	20.98	0.30		Genotype	0.0430
Nitrogen		Yes	10	23.17	1.36		28	24.54	0.81		44	21.45	0.65		Stones	0.0003
(mg/dL)															Gen*Stones	0.0115
																
Creatinine		No	19	1.190	0.066		137	1.198	0.025		200	1.238	0.020		Genotype	0.3360
(mg/dL)		Yes	10	1.368	0.092		28	1.410	0.055		44	1.257	0.044		Stones	0.0029
															Gen*Stones	0.0351
																
Cholesterol		No	19	145.4	11.0		137	149.4	4.1		201	161.9	3.4		Genotype	0.0932
(mg/dL)		Yes	10	158.2	15.1		28	168.5	9.0		44	177.5	7.2		Stones	0.0364
															Gen*Stones	0.9393
																
Glucose		No	19	95.21	3.82		136	99.63	1.43		201	97.33	1.18		Genotype	0.4795
(mg/dL)		Yes	10	110.40	5.27		28	99.50	3.15		44	99.77	2.51		Stones	0.0268
															Gen*Stones	0.1144
																
Inorganic		No	19	4.44	0.26		137	4.09	0.10		201	3.82	0.08		Genotype	0.7117
Phosphorus		Yes	10	3.42	0.36		28	3.61	0.21		44	3.70	0.17		Stones	0.0028
(mg/dL)															Gen*Stones	0.1284
																
Magnesium		No	19	2.011	0.032		137	2.031	0.012		200	2.028	0.010		Genotype	0.6190
(mg/dL)		Yes	10	2.010	0.044		28	2.021	0.026		44	1.989	0.021		Stones	0.4629
															Gen*Stones	0.6546
																
Sodium		No	19	152.95	0.39		137	153.01	0.14		201	153.35	0.12		Genotype	0.3787
(mmol/L)		Yes	10	153.10	0.53		28	153.54	0.32		44	153.59	0.25		Stones	0.2483
															Gen*Stones	0.7768
																
SDMA		No	19	14.46	0.65		136	12.73	0.24		201	12.09	0.20		Genotype	<.0001
(µg/dL)		Yes	10	16.05	0.90		28	14.56	0.54		44	12.36	0.43		Stones	0.0060
															Gen*Stones	0.0972
																
Total Protein		No	19	6.78	0.13		137	6.86	0.05		201	6.88	0.04		Genotype	0.4881
(g/dL)		Yes	10	6.99	0.19		28	7.18	0.11		44	7.02	0.09		Stones	0.0171
															Gen*Stones	0.5473
																
Hemoglobin		No	19	11.55	0.37		135	11.09	0.14		200	11.12	0.11		Genotype	0.8211
(g/dL)		Yes	10	10.85	0.50		28	10.91	0.30		44	10.87	0.24		Stones	0.1305
															Gen*Stones	0.7593
																
HCT		No	19	34.95	1.13		135	33.71	0.42		200	33.89	0.35		Genotype	0.9276
(%)		Yes	10	32.24	1.56		28	32.68	0.93		44	32.83	0.74		Stones	0.0400
															Gen*Stones	0.7141
																
MCV		No	19	43.13	0.89		135	43.43	0.33		200	43.84	0.27		Genotype	0.0371
(fL)		Yes	10	39.52	1.22		28	42.60	0.73		44	43.03	0.58		Stones	0.0044
															Gen*Stones	0.2163
																
MCH		No	19	14.25	0.26		135	14.29	0.10		200	14.37	0.08		Genotype	0.0786
(pg)		Yes	10	13.28	0.36		28	14.21	0.22		44	14.25	0.17		Stones	0.0325
															Gen*Stones	0.1844
																
MCHC		No	19	33.09	0.35		135	32.95	0.13		200	32.85	0.11		Genotype	0.4081
(g/dL)		Yes	10	33.68	0.49		28	33.43	0.29		44	33.15	0.23		Stones	0.0609
															Gen*Stones	0.8673
																
Absolute		No	19	0.068	0.006		135	0.061	0.002		200	0.061	0.002		Genotype	0.0230
Reticulocytes		Yes	10	0.075	0.009		28	0.066	0.005		44	0.053	0.004		Stones	0.7742
(M/µL)															Gen*Stones	0.1411
																
Neutrophils		No	19	55.95	3.00		132	58.82	1.14		198	59.41	0.93		Genotype	0.0407
(%)		Yes	10	55.16	4.14		28	65.71	2.47		44	58.75	1.97		Stones	0.3806
															Gen*Stones	0.0827

**Table 3:** (**A**) Serum metabolites that varied significantly among the three genotypes in cats with different AGXT2 SNPs from a GWAS of 445 cats in the colony at Hill’s Pet Nutrition, Inc. (**B**) Factor analysis was used as a data-reduction method for the metabolites (see Section 2.5).

(**A**)												
**Factor 1**							**Factor 2**					
**Metabolite**					**Loading**		**Metabolite**					**Loading**
sphingomyelin (d18:2/23:0, d18:1/23:1, d17:1/24:1)					0.9119		decanoylcarnitine (C10)					0.8832
Cholesterol					0.8531		myristoylcarnitine (C14)					0.8601
sphingomyelin (d18:1/21:0, d17:1/22:0, d16:1/23:0)					0.8497		octanoylcarnitine (C8)					0.8477
tricosanoyl sphingomyelin (d18:1/23:0)					0.8477		hexanoylcarnitine (C6)					0.8219
sphingomyelin (d18:1/15:0, d16:1/17:0)					0.8413		eicosenoylcarnitine (C20:1)					0.8184
palmitoyl sphingomyelin (d18:1/16:0)					0.8260		dihomo-linoleoylcarnitine (C20:2)					0.8181
sphingomyelin (d18:1/19:0, d19:1/18:0)					0.8243		laurylcarnitine (C12)					0.8166
sphingomyelin (d18:2/21:0, d16:2/23:0)					0.7929		palmitoylcarnitine (C16)					0.8026
sphingomyelin (d17:2/16:0, d18:2/15:0)					0.7905		myristoleoylcarnitine (C14:1)					0.8021
sphingomyelin (d18:2/23:1)					0.7898		oleoylcarnitine (C18)					0.7998
sphingomyelin (d18:2/16:0, d18:1/16:1)					0.7851		palmitoleoylcarnitine (C16:1)					0.7941
sphingomyelin (d18:1/17:0, d17:1/18:0, d19:1/16:0)					0.7634		margaroylcarnitine					0.7794
1-lignoceroyl-GPC (24:0)					0.7593		stearoylcarnitine (C18)					0.7793
behenoyl sphingomyelin (d18:1/22:0)					0.7351		linoleoylcarnitine (C18:2)					0.7712
glycosyl ceramide (d18:2/24:1, d18:1/24:2)					0.7164		3-hydroxybutyrylcarnitine (1)					0.7601
sphingomyelin (d18:1/20:0, d16:1/22:0)					0.7090		acetylcarnitine (C2)					0.7305
sphingomyelin (d18:1/22:1, d18:2/22:0, d16:1/24:1)					0.7009		butyrylcarnitine (C4)					0.7274
S-methylcysteine					−0.2027		erucoylcarnitine (C22:1)					0.7137
4-imidazoleacetate					−0.2379		cis-4-decenoylcarnitine (C10:1)					0.7053
							pyridoxal					−0.3106
**Factor 3**							2-hydroxyglutarate					−0.3128
**Metabolite**					**Loading**		phosphate					−0.3145
N6-carbamoylthreonyladenosine					0.8181		N-acetylfelinine					−0.3149
pseudouridine					0.7937		γ-glutamylfelinylglycine					−0.3196
allantoin					0.7706		hydroxyproline					−0.3732
N1-methylinosine					0.7678		1-(1-enyl-palmitoyl)-2-oleoyl-GPE (P-16:0/18:1)					−0.3791
C-glycosyltryptophan					0.7576							
N-acetyltaurine					0.7413		**Factor 5**					
allantoic acid					0.7349		**Metabolite**					**Loading**
erythronate					0.7086		linoleate (18:2n6)					0.9026
7-methylguanine					0.7025		oleate/vaccenate (18:1)					0.8998
erythritol					0.6829		palmitate (16:0)					0.8854
N-acetylthreonine					0.6809		stearate (18:0)					0.8794
O-sulfo-L-tyrosine					0.6443		10-nonadecenoate (19:1n9)					0.8764
dimethylarginine (ADMA + SDMA)					0.6414		eicosenoate (20:1n9 or 1n11)					0.8546
1-methylhistidine					0.6387		margarate (17:0)					0.8518
N-acetylserine					0.6383		palmitoleate (16:1n7)					0.8350
kynurenate					0.6111		10-heptadecenoate (17:1n7)					0.8335
docosadioate					−0.3012		dihomolinolenate (20:3n3 or 3n6)					0.8285
threonine					−0.3303		dihomolinoleate (20:2n6)					0.8240
							nonadecanoate (19:0)					0.8173
**Factor 9**							p-cresol sulfate					−0.2573
**Metabolite**					**Loading**		tryptophan					−0.2587
suberate (octanedioate)					0.9364		hypotaurine					−0.2821
azelate (nonanedioate; C9)					0.9295							
dodecanedioate (C12)					0.9255		**Factor 12**					
sebacate (decanedioate)					0.9248		**Metabolite**					**Loading**
undecanedioate					0.9197		pyruvate					0.7949
1,11-undecanedicarboxylate					0.9192		α-ketoglutarate					0.7825
8-hydroxyoctanoate					0.9035		lactate					0.7563
pimelate (heptanedioate)					0.8859		fumarate					0.7077
adipate					0.8646		malate					0.7055
pelargonate (9:0)					0.8434		3-methyl-2-oxobutyrate					0.6306
tetradecanedioate (C14)					0.7882		alanine					0.6271
5-hydroxyhexanoate					0.7306		2-oxoadipate					0.5845
glutarate (pentanedioate)					0.7067		4-methyl-2-oxopentanoate					0.5525
1-palmitoyl-2-oleoyl-GPI (16:0/18:1)					−0.1730		3-methyl-2-oxovalerate					0.5372
1-stearoyl-GPI (18:0)					−0.1774		N-acetylglutamine					0.5263
							2-hydroxy-3-methylvalerate					0.5207
							4-guanidinobutanoate					−0.4604
**Factor 18**							**Factor 20**					
**Metabolite**					**Loading**		**Metabolite**					**Loading**
cysteine sulfinic acid					0.5742		γ-glutamylfelinylglycine					0.3975
benzoate					0.5623		sulfate					0.3684
heme					0.5429		7-hydroxyindole sulfate					0.3679
biliverdin					0.3969		cysteine s-sulfate					0.3487
cortisone					0.3705		homocitrulline					0.3473
pyridoxal					0.3523		isovalerylglycine					0.3354
2’-deoxyuridine					0.3406		2-oxoarginine					0.3325
glycerol					0.3041		4-guanidinobutanoate					0.3162
thioproline					−0.2927		fumarate					0.3140
maltose					−0.3395		dimethylmalonic acid					0.3140
mannitol/sorbitol					−0.4998		malate					0.3108
							N-linolenoyltaurine					−0.3460
(**B**)												
**Analyte**	**Stones**	**Genotype = AA**			**Genotype = AG**			**Genotype = GG**			***p*-Values**	
	**Present**	**n**	**Mean**	**SE**	**n**	**Mean**	**SE**	**n**	**Mean**	**SE**	**Source**	**Prob > F**
Factor 1	No	20	−0.43	0.22	139	−0.08	0.08	204	−0.02	0.07	Genotype	0.0740
	Yes	10	−0.14	0.31	28	0.28	0.19	44	0.41	0.15	Stones	0.0202
											Gen*Stones	0.9247
Factor 2	No	20	−0.12	0.22	139	−0.18	0.08	204	0.15	0.07	Genotype	0.0416
	Yes	10	−0.32	0.31	28	−0.16	0.19	44	0.11	0.15	Stones	0.6513
											Gen*Stones	0.8801
Factor 3	No	20	0.14	0.22	139	−0.10	0.08	204	−0.08	0.07	Genotype	0.0144
	Yes	10	0.66	0.31	28	0.70	0.19	44	0.02	0.15	Stones	0.0024
											Gen*Stones	0.0273
Factor 5	No	20	0.39	0.22	139	0.16	0.08	204	−0.07	0.07	Genotype	0.4347
	Yes	10	−0.31	0.31	28	−0.15	0.19	44	−0.22	0.15	Stones	0.0128
											Gen*Stones	0.4084
Factor 9	No	20	0.42	0.22	139	−0.08	0.08	204	0.09	0.07	Genotype	0.6422
	Yes	10	−0.34	0.31	28	−0.19	0.19	44	−0.16	0.15	Stones	0.0153
											Gen*Stones	0.3264
Factor 12	No	20	0.09	0.22	139	0.04	0.08	204	−0.12	0.07	Genotype	0.0533
	Yes	10	0.92	0.31	28	0.12	0.19	44	0.11	0.15	Stones	0.0151
											Gen*Stones	0.2376
Factor 18	No	20	−0.42	0.22	139	0.06	0.08	204	0.03	0.07	Genotype	0.0072
	Yes	10	−0.75	0.31	28	−0.17	0.19	44	0.11	0.15	Stones	0.2949
											Gen*Stones	0.3928
Factor 20	No	20	−0.28	0.22	139	−0.09	0.08	204	0.05	0.07	Genotype	0.0006
	Yes	10	−0.40	0.31	28	−0.30	0.19	44	0.43	0.15	Stones	0.9375
											Gen*Stones	0.0655

**Table 4:** Variables selected for discriminating among the AA, AG, and GG genotypes.

Step	Entered	Partial R^2^	F Value	Pr > F
1	SDMA	0.0726	16.87	<0.0001
2	Cholesterol	0.0234	5.15	0.0062
3	Factor 3	0.0188	4.11	0.0171
4	Factor 12	0.0204	4.46	0.0122
5	Factor 18	0.0177	3.86	0.0219
6	Total Bilirubin	0.0146	3.15	0.0439
7	Factor 1	0.0158	3.41	0.0341
8	Factor 20	0.0137	2.94	0.0537
9	Factor 2	0.0118	2.52	0.0820
10	Factor 5	0.0107	2.28	0.1038
11	Glucose	0.0104	2.21	0.1109

**Table 5:** Variables selected for discriminating between stone-formers and non-stone formers.

Step	Entered	Partial R^2^	F Value	Pr > F
1	Factor 3	0.0260	11.53	0.0007
2	ALP	0.0205	9.01	0.0028
3	MCHC	0.0233	10.24	0.0015
4	Factor 1	0.0160	6.97	0.0086
5	Total Protein	0.0120	5.20	0.0230
6	Magnesium	0.0116	5.02	0.0255
7	Sodium	0.0103	4.45	0.0355

## Data Availability

All relevant data are within the paper and its Supplementary Information files.

## Supplementary Materials

The following supporting information can be downloaded at: https://www.mdpi.com/article/10.3390/genes15101264/s1, Table S1. The mean scaled imputed values for all untargeted biochemicals when the presence or absence of stones was assessed relative to the genotype for different AGXT2 SNPs (AA, AG, GG) from a GWAS of 445 cats in the colony at Hill’s Pet Nutrition, Inc. Table S2: Factor analysis was used as a data-reduction method for the more than 600 serum metabolites included in the analysis because many of these metabolites are correlated. Principal component analysis was used as the method of factor extraction. The resulting patterns were then rotated using a varimax rotation to aid interpretation. The advantage of a varimax rotation is all the factors remain orthogonal (independent) and each metabolite tends to load highly on only one factor, making it easier to interpret the factor patterns. Factor analysis was performed on the correlation matrix so that unequal variances among the metabolites did not unduly influence the resulting factor patterns. Using the correlation matrix, each metabolite has a variance of 1. To reduce the number of factors that needed to be interpreted, only factors with eigenvalues greater than 6 were retained for rotation. An eigenvalue of 6 indicates that a factor accounts for at least 1% of the total variation in the data. Using this approach, the 600 serum metabolites were reduced to 20 factors. The resulting factors were then analyzed using ANOVA with genotype, stones, and the interaction as fixed-effects in the model. Factor means are linear combinations of all metabolites, with higher coefficients or weights for metabolites that load strongly on that factor and small coefficients or weights for metabolites that are only weakly or not associated with that factor. Because the serum metabolic data are median-normalized, a negative mean indicates that the mean level of the metabolic factor is below the median, and a positive mean indicates that the mean level of that factor is above the median. Table S3A: Demographics and stone analysis for 81 cats forming stones from a GWAS of 445 cats in the colony at Hill’s Pet Nutrition, Inc. Table S3B: Demographics for 364 cats (non-stone formers) from a GWAS of 445 cats in the colony at Hill’s Pet Nutrition, Inc. Table S4: CBC and serum biochemistry analytes in cats with different AGXT2 SNPs from a GWAS of 445 cats in the colony at Hill’s Pet Nutrition, Inc. Table S5: All serum metabolite factor statistical results in cats with different AGXT2 SNPs from a GWAS of 445 cats in the colony at Hill’s Pet Nutrition, Inc.
